# Risk of recurrent stroke and antiplatelet choice in breakthrough stroke while on aspirin

**DOI:** 10.1038/s41598-020-73836-0

**Published:** 2020-10-07

**Authors:** Joon-Tae Kim, Beom Joon Kim, Jong-Moo Park, Soo Joo Lee, Jae-Kwan Cha, Tai Hwan Park, Kyung Bok Lee, Jun Lee, Keun-Sik Hong, Byung-Chul Lee, Dong-Eog Kim, Jay Chol Choi, Jee-Hyun Kwon, Dong-Ick Shin, Sung Il Sohn, Ji Sung Lee, Juneyoung Lee, Hee-Joon Bae

**Affiliations:** 1 Department of Neurology, Chonnam National University Hospital, Chonnam National University Medical School, Gwangju, Republic of Korea; 2 Department of Neurology, Cerebrovascular Center, Seoul National University College of Medicine, Seoul National University Bundang Hospital, 82, Gumi-ro 173 beon-gil, Bundang-gu, Seongnam-si, Gyeonggi-do 13620 Republic of Korea; 3 Department of Neurology, Eulji General Hospital, Eulji University, Seoul, Republic of Korea; 4 Department of Neurology, Eulji University Hospital, Eulji University, Daejeon, Republic of Korea; 5 Department of Neurology, Dong-A University Hospital, Busan, Republic of Korea; 6 Department of Neurology, Seoul Medical Center, Seoul, Republic of Korea; 7 Department of Neurology, Soonchunhyang University Hospital, Seoul, Republic of Korea; 8 Department of Neurology, Yeungnam University Hospital, Daegu, Republic of Korea; 9 Department of Neurology, Ilsan Paik Hospital, Inje University, Goyang, Republic of Korea; 10 Department of Neurology, Hallym University Sacred Heart Hospital, Anyang, Republic of Korea; 11 Department of Neurology, Dongguk University Ilsan Hospital, Goyang, Republic of Korea; 12 Department of Neurology, Jeju National University Hospital, Jeju National University School of Medicine, Jeju, Republic of Korea; 13 Department of Neurology, Ulsan University College of Medicine, Ulsan, Republic of Korea; 14 Department of Neurology, Chungbuk National University Hospital, Cheongju, Republic of Korea; 15 Department of Neurology, Keimyung University Dongsan Medical Center, Daegu, Republic of Korea; 16 Clinical Research Center, Asan Medical Center, Seoul, Republic of Korea; 17 Department of Biostatistics, Korea University College of Medicine, Seoul, Republic of Korea

**Keywords:** Neurology, Cerebrovascular disorders, Stroke

## Abstract

Uncertainty regarding an optimal antiplatelet regimen still exists in patients with breakthrough acute ischemic stroke (AIS) while on aspirin. This study provides an analysis of a prospective multicenter registry between April 2008 and April 2014. Eligible patients were on aspirin at the time of AIS and treated with antiplatelet regimens (aspirin, clopidogrel, or clopidogrel-aspirin). Potential factors associated with the choice of each antiplatelet regimen were explored and included a predictive risk score for future vascular events, the Essen Stroke Risk Score (ESRS). A total of 2348 patients (age, 69 ± 11 years; male, 57.7%) were analyzed, and 55.3%, 25.3% and 19.4% were treated with clopidogrel-aspirin, aspirin and clopidogrel, respectively. While the likelihood of choosing clopidogrel-aspirin increased as the ESRS increased, the likelihood of choosing aspirin decreased as the ESRS increased (P_trend_ < 0.001). The ESRS category (0–1/2–3/ ≥ 4) modified the effect of antiplatelet regimens for 1-year vascular events (P_interaction_ < 0.01). Among patients with ESRS ≥ 4, clopidogrel-aspirin (HR 0.47 [0.30–0.74]) and clopidogrel (HR 0.30 [0.15–0.60]) significantly reduced the risk of outcome events. Our study showed that more than half of the patients with aspirin failure were treated with clopidogrel-aspirin. In particular, a higher ESRS, which indicates an increased risk of recurrent stroke, was associated with the choice of clopidogrel-aspirin rather than aspirin.

## Introduction

Aspirin has been considered a first-line antiplatelet strategy for the secondary prevention of ischemic stroke or transient ischemic attack (TIA)^1^. Clopidogrel and a combination of aspirin and extended-release dipyridamole (ER-DP) are also indicated as initial therapies for the prevention of subsequent stroke in patients who have experienced stroke^1^. A combination of aspirin and clopidogrel may be considered in specific conditions, such as angioplasty and stenting for extracranial carotid stenosis for a limited period, minor stroke or TIA within 24 h of onset during the first 90 days of stroke, or symptomatic severe intracranial stenosis with expectation of more potent antiplatelet effects^2,3^. Although substantial progress has been made in the prevention and treatment of stroke, in most circumstances, stroke guidelines generally leave the selection of antiplatelet regimen to the treating physicians.

Because the use of aspirin for primary prevention of stroke and cardiovascular disease is increasing^4^, physicians have encountered an increasing number of patients who have experienced breakthrough stroke or TIA while taking aspirin, which is termed ‘aspirin failure’. Recent studies have reported that switching to or adding another antiplatelet agent in these patients yielded better prevention of subsequent vascular events after breakthrough stroke while on aspirin^5–7^. However, uncertainty still exists for an optimal antiplatelet regimen in this clinical circumstance.

Exploring the association of selected antiplatelet regimens and clinical factors in patients with breakthrough stroke while on aspirin might help to determine physicians’ behavior in a clinical circumstance where evidence is lacking. Estimations of risk for recurrent stroke might be helpful in determining the most beneficial treatment among various therapeutic options. The Essen Stroke Risk Score (ESRS) is a 10-point scale derived and validated from the datasets of large clinical trials, and it can help physicians predict the 1-year risk of recurrent stroke and cardiovascular events in patients with acute ischemic stroke^8–11^.

This study aimed to explore the factors associated with the choice of antiplatelet regimen and elucidate the associations between the choice of antiplatelet regimen and the ESRS in breakthrough stroke while on aspirin. We hypothesized that a higher ESRS might be associated with a more potent antiplatelet regimen, such as combination of aspirin and clopidogrel, and additionally investigated whether the effects of these different antiplatelet regimens on the prevention of subsequent vascular events were modified by the ESRS categories.

## Methods

### Subjects

This study was performed through an analysis of the Clinical Research Center for Stroke-5th division (CRCS-5) registry, a prospective, nationwide, multicenter, acute stroke registry database that was established in 2008. Detailed information on the CRCS-5 registry has been previously reported^12,13^. Between April 2008 and April 2014, 30,671 acute stroke patients were treated by 74 neurologists in 14 participating centers. Among those, we selected patients who met the following eligibility criteria for this study: (1) acute ischemic stroke or TIA within 7 days of onset, (2) non-cardioembolic stroke, and (3) already taking aspirin monotherapy for 7 days or more prior to the index acute ischemic stroke or TIA. We excluded patients (1) who had potential sources of cardioembolism, such as atrial fibrillation, (2) who took oral anticoagulants during admission or at discharge, and (3) who were not on antiplatelet treatment at discharge. Among the various antithrombotic strategies, the 3 most common antiplatelet regimens in South Korea^5,12^ (aspirin monotherapy [AM], clopidogrel monotherapy [CM], or combination of aspirin and clopidogrel [AC]) were selected as antiplatelet regimens of interest for this study. In Korea, dual antiplatelet therapy with aspirin plus dipyridamole was not commercially available during the study period.

### Ethics statements

The collection of clinical information for the purposes of monitoring and improving the quality and outcomes of stroke care was approved by the Institutional Review Boards (IRBs) of Seoul National University Bundang Hospital (SNUBH) and other participating centers of the CRCS-5 registry with a waiver of consent because of the study patients’ anonymity and minimal risk to the patients. Use of the registry database and an additional review of medical records for the present study were also approved by the individual IRBs. We confirm that all methods were performed in accordance with the principles expressed in the Declaration of Helsinki.

### Data collection

Demographic, clinical, imaging, and laboratory data were prospectively collected. The following data were directly obtained from the registry database: (1) demographics: age and sex; (2) vascular risk factors: hypertension, diabetes mellitus, dyslipidemia, current smoking, history of coronary artery diseases (CAD), history of stroke or TIA, and history of peripheral artery diseases (PAD); (3) stroke characteristics and acute treatment: initial National Institutes of Health Stroke Scale (NIHSS) score, prestroke modified Rankin Scale (mRS) score, ischemic stroke subtype according to the Trial of Org 10,172 in Acute Stroke Treatment (TOAST) criteria after complete diagnostic profiling^14,15^, relevant cerebral artery diseases (RAD) (defined as stenosis > 50% and occlusion) and thrombolytic therapy; and (4) prior statin use and discharge medications of statins, antihypertensive agents, and antidiabetics. The ESRS was retrospectively calculated as the sum score (0–9 points) based on the following risk factors: 2 points for age > 75 years, 1 point each for age 65–75 years, arterial hypertension, diabetes mellitus, previous myocardial infarction, other cardiovascular diseases (except myocardial infarction and atrial fibrillation), peripheral arterial disease, smoking, and previous TIA or ischemic stroke in addition to the qualifying event (Supplemental Table 1)^8^. On a 10-point scale, the ESRS predicts the 1-year risk of recurrent stroke and combined cardiovascular events, with higher scores relating to higher risk of vascular events. To investigate the different effects of antiplatelet regimen according to the ESRS categories, the ESRS was arbitrarily categorized into 3 groups; 0–1, 2–3, and 4 or more.

**Table 1:** Comparisons of patient characteristics according to antiplatelet regimen.

	AM group	CM group	AC group	P
N	593	456	1299	
Age	66 ± 12	70 ± 11	69 ± 11	< 0.001
Male	334 (56.3)	252 (55.3)	769 (59.2)	0.17
Time to admission within 24 h	403 (68.0)	285 (62.5)	783 (60.3)	0.002
Prestroke mRS > 1	92 (15.5)	74 (16.2)	198 (15.2)	0.82
Baseline NIHSS (med, IQR)	2 (0, 5)	2 (1, 4)	3 (1, 5)	0.02
Presenting event				0.001
TIA	116 (19.6)	58 (12.7)	171 (13.2)	
Ischemic stroke	477 (80.4)	398 (87.3)	1128 (86.8)	
TOAST (except TIA)				< 0.001
LAA	196 (41.1)	159 (39.9)	608 (53.9)	
SVO	132 (27.7)	161 (40.5)	273 (24.2)	
OE	15 (3.1)	6 (1.5)	20 (1.8)	
UD	134 (28.1)	72 (18.1)	227 (20.1)	
History of TIA	18 (3.0)	21 (4.6)	64 (4.9)	0.07
History of stroke	147 (24.8)	165 (36.2)	391 (30.1)	0.08
History of PAD	5 (0.8)	2 (0.4)	13 (1.0)	0.60
History of CAD	63 (10.6)	55 (12.1)	240 (18.5)	< 0.001
CAD except MI	36 (6.1)	38 (8.3)	153 (11.8)	< 0.001
MI	27 (4.6)	17 (3.8)	87 (6.7)	0.03
HTN	450 (75.9)	393 (86.2)	1126 (86.7)	< 0.001
DM	212 (35.8)	205 (45.0)	583 (44.9)	0.001
Dyslipidemia	186 (31.4)	224 (49.1)	504 (38.8)	0.03
Smoking	160 (27.0)	111 (24.3)	328 (25.3)	0.50
Prior statin	159 (26.8)	138 (30.3)	408 (31.4)	0.05
Prior antihypertensive	396 (66.8)	366 (80.3)	1082 (83.3)	< 0.001
Prior antidiabetics	173 (29.2)	179 (39.3)	491 (37.8)	0.001
RAD (> 50%)	186 (31.4)	125 (27.4)	542 (41.7)	< 0.001
Laboratory findings				
White blood cells	8.17 (4.39)	7.70 (2.83)	8.03 (2.87)	0.06
Hemoglobin (mg/dl)	13.5 (2.05)	13.3 (1.87)	13.50 (1.80)	0.15
Glucose (mg//dl)	141 ± 70	145 ± 72	142 ± 64	0.61
LDL (mg/dl)	104 ± 36	109 ± 36	103 ± 33	0.006
SBP (mmHg)	148 ± 27	145 ± 25	149 ± 25	0.009
Reperfusion therapy	41 (6.9)	33 (7.2)	94 (7.2)	0.81
IV only	29 (4.9)	26 (5.7)	60 (4.6)	
IA only	5 (0.8)	5 (1.1)	21 (1.6)	
IV + IA	7 (1.2)	2 (0.4)	13 (1.0)	
Hospital treatment				
Antihypertensive	279 (47.0)	265 (58.1)	728 (56.0)	0.001
Antidiabetics	157 (36.5)	167 (36.6)	453 (34.9)	0.001
Statin	451 (76.1)	368 (80.7)	1156 (89.0)	< 0.001
ESRS (med, IQR)	3 (2, 4)	3 (2, 5)	3 (2, 4)	< 0.001
1 or more	570 (96.1)	454 (99.6)	1288 (99.2)	< 0.001
2 or more	496 (83.6)	421 (92.3)	1221 (94.0)	< 0.001

*P*; comparisons among 3 groups.

Abbreviations: AM; aspirin monotherapy, CM; clopidogrel monotherapy, AC; combination therapy of aspirin and clopidogrel, mRS; modified Rankin Scale, NIHSS; National Institutes of Health Stroke Scale, TIA; transient ischemic attack, TOAST; Trials of Org 10,172 in Acute Stroke Treatment, LAA; large artery atherosclerosis, SVO; small vessel occlusion, OE; other etiology, UD; undetermined etiology, PAD; peripheral artery disease, CAD; coronary artery disease, MI; myocardial infarction, HTN; hypertension, DM; diabetes mellitus, RAD; relevant cerebral artery disease, ESRS; Essen Stroke Risk Score.

### Outcome measurement

Using a predefined protocol from the CRCS-5 registry^12,13^, we prospectively captured vascular events during follow-up at 3 months and 1 year after the qualifying event during routine clinic visits or by telephone interviews with patients or their caregivers as previously described^26^. To assure the accuracy of the outcome record and minimize the difference of the outcome capture process according to the interviewers, a set of uniform structured questionnaires was used and regular education was provided for the interviewers. The primary outcome was a composite of stroke, myocardial infarction (MI), or all-cause mortality up to 1 year after stroke.

### Statistical analyses

We compared the baseline characteristics of patients according to the 3 selected antiplatelet regimens: AM, CM, and AC. The frequencies (%), means ± SDs, or medians (interquartile ranges, IQRs) were reported depending on variable characteristics. Categorical variables were compared using the χ2-test or Fisher’s exact test as appropriate. Continuous variables were compared using the analysis of variance test or the Kruskal–Wallis test as appropriate. To identify determinants of antiplatelet regimens, multivariate logistic regression analysis using generalized linear mixed models to account for the center effect (using a random intercept model) was performed. The following variables were included in the models based on prior literatures and clinical relevance: age, gender, onset to admission, initial NIHSS score, prestroke disability of mRS score, TOAST classification, prior TIA, prior stroke, prior PAD, prior myocardial infarction, prior CAD (except myocardial infarction), hypertension, diabetes, dyslipidemia, smoking, prior statin use, prior antihypertensive use, prior antidiabetics use, RAD, thrombolysis, anti-diabetes treatment, antihypertensive treatment, statin treatment, systolic blood pressure (SBP), glucose, and low-density lipoprotein (LDL). To explore the associations between the ESRS and the 3 antiplatelet regimens, the ESRS was analyzed as both binary (ESRS 0 vs ≥ 1, 0–1 vs ≥ 2, and 0–2 vs ≥ 3) and continuous (every 1-point increase of ESRS) variables. Variables for adjustment were predetermined based on prior studies and clinical relevance: sex, NIHSS, onset to visit time (within 24 h vs > 24 h), premorbid disability (prestroke mRS 0–1 vs > 1), dyslipidemia, RAD, prior statin use, and TOAST classifications. Predetermined subgroup analyses were performed according to the following factors: initial NIHSS score (≤ 4 vs > 4), onset to admission (≤ 24 h vs > 24 h), RAD, and TOAST classifications. Event rates of 1-year primary composite outcome measures were estimated using Kaplan–Meier product-limit method and were also compared among the AM, CM, and AC groups by log-rank test. Cox proportional hazards regression analysis was used to evaluate the independent effects of antiplatelet regimen modifications on outcome events. Adjustments were made for predetermined variables whose associations with outcome variables were clinically relevant; age, sex, NIHSS, ischemic events subtype (TOAST including TIA), RAD, prior statin use, and the ESRS. Hazard ratios and 95% confidence intervals were estimated. To explore the existence of effect modifications by the ESRS, an interaction term between antiplatelet therapy regimen and 3 ESRS categories (ESRS; 0–1/2–3/4 or more) was generated, and its statistical significance was examined using the Cox proportional hazards models. The strength of the associations was estimated using odds ratios (ORs) and 95% confidence intervals (CIs). Statistical significance was determined via a 2-tailed P-value of < 0.05. All statistical analyses were performed using SPSS for Windows version 17 (SPSS Inc., Chicago, IL, USA) and SAS (version 9.4; SAS Institute, Cary, NC, USA).

## Results

### General characteristics

Of the 30,135 patients with acute cerebral ischemia registered between April 2008 and March 2014, 3,140 met the eligibility criteria of non-cardioembolic breakthrough stroke while on aspirin. Among those, 792 patients were excluded: 446 due to oral anticoagulant use during hospitalization or at discharge and 346 due to antiplatelet regimens other than AM, CM, and AC (Supplemental Figure I). Ultimately, 2,348 patients (mean age, 69 ± 11 years; males, 57.7%) were included in this study. The median ESRS of the all study subjects was 3 (IQR 2, 4).

Table 1 shows the patient characteristics according to the 3 common antiplatelet regimens. AM was used in 593 (25.3%) patents, CM in 456 (19.4%), and AC in 1,299 (55.3%), and the median ESRSs of the 3 groups were 3 (IQR, 2 to 4), 4 (2 to 5), and 3 (2 to 4), respectively (P < 0.001). The characteristics of 346 patients who received other antiplatelet regimens and were excluded are summarized in Supplemental Tables II. The detailed antiplatelet regimens after new ischemic stroke are shown in Supplemental Table III.

### Independent factors associated with the choice of antiplatelet regimens

Unadjusted and adjusted analyses indicated that the independent factors associated with CM use versus AM use were older age, history of stroke, dyslipidemia, prior antihypertensive use, and SVO (Table 2 and Table 3). Patients with a higher NIHSS score were less likely to receive CM. Independent factors associated with taking AC compared with taking AM were older age, LAA, history of TIA, history of stroke, history of CAD, prior antihypertensive use, RAD, and statin treatment. Patients with higher NIHSS scores, TIA at presentation, and prior statin use were less likely to be treated with AC. Compared with CM use, AC use was independently associated with male sex, history of CAD, RAD, statin treatment during hospitalization, and higher SBP. In contrast, SVO and dyslipidemia were associated with a lower probability of taking AC (Table 2).

**Table 2:** Potential factors associated with taking each antiplatelet.

	CM (vs AM)			AC (vs AM)			AC (vs CM)		
	aOR (95% CI)		P	aOR (95% CI)		P	aOR (95% CI)		P
Age, 10 years	1.37	(1.16–1.62)	0.0002	1.22	(1.10–1.36)	0.0003	0.93	(0.80–1.07)	0.30
Male	0.96	(0.67–1.37)	0.80	1.26	(0.98–1.62)	0.07	1.43	(1.05–1.94)	0.023
Onset to arrival									
Within 24 h	0.87	(0.62–1.23)	0.43	0.85	(0.67–1.08)	0.18	0.78	(0.59–1.04)	0.09
Initial NIHSS	0.94	(0.91–0.98)	0.002	0.95	(0.93–0.98)	0.001	1.01	(0.97–1.05)	0.59
Pre-mRS > 1	0.71	(0.44–1.14)	0.16	1.01	(0.72–1.42)	0.95	1.23	(0.83–1.83)	0.31
Mechanisms									
TIA	0.70	(0.40–1.22)	0.21	0.66	(0.46–0.97)	0.03	0.78	(0.47–1.30)	0.34
LAA	1.27	(0.80–2.00)	0.31	1.60	(1.17–2.18)	0.003	1.19	(0.80–1.78)	0.39
SVO	1.78	(1.11–2.87)	0.02	1.36	(0.97–1.90)	0.08	0.61	(0.40–0.93)	0.02
OE/UD (ref)	Ref			Ref			Ref		
History of TIA	2.04	(0.88–4.72)	0.10	1.84	(1.01–3.38)	0.05	1.34	(0.71–2.51)	0.37
History of stroke	1.58	(1.12–2.24)	0.01	1.56	(1.20–2.04)	0.001	0.82	(0.61–1.11)	0.20
History of PAD	0.85	(0.15–4.93)	0.86	1.19	(0.35–4.11)	0.78	1.27	(0.22–7.20)	0.79
History of CAD	1.06	(0.64–1.75)	0.82	1.70	(1.21–2.40)	0.003	1.80	(1.22–2.66)	0.003
HTM	0.68	(0.37–1.27)	0.23	0.95	(0.61–1.46)	0.80	0.76	(0.42–1.36)	0.36
DM	0.69	(0.34–1.41)	0.31	1.02	(0.65–1.61)	0.93	1.17	(0.65–2.12)	0.61
Dyslipidemia	1.94	(1.30–2.89)	0.001	1.30	(0.97–1.76)	0.08	0.64	(0.45–0.91)	0.01
Smoking, current	1.19	(0.79–1.78)	0.40	0.94	(0.71–1.25)	0.68	1.06	(0.75–1.50)	0.74
Prior statin	0.75	(0.48–1.18)	0.21	0.71	(0.52–0.98)	0.04	1.34	(0.91–1.99)	0.14
Prior antihypertensive	1.75	(1.02–3.01)	0.04	1.94	(1.32–2.84)	0.001	1.41	(0.85–2.34)	0.19
Prior anti-diabetes	1.65	(0.85–3.19)	0.14	1.06	(0.68–1.65)	0.79	0.70	(0.41–1.22)	0.21
RAD	0.86	(0.57–1.30)	0.47	1.41	(1.07–1.84)	0.01	1.58	(1.11–2.23)	0.01
Thrombolysis	1.64	(0.85–3.15)	0.14	1.32	(0.82–2.12)	0.25	0.72	(0.41–1.26)	0.25
Anti-diabetes Tx	0.93	(0.57–1.52)	0.77	1.40	(0.98–1.98)	0.06	1.08	(0.70–1.66)	0.72
Antihypertensive Tx	1.29	(0.91–1.81)	0.15	1.00	(0.79–1.27)	1.00	0.84	(0.62–1.13)	0.25
Statin treatment	0.85	(0.55–1.30)	0.46	2.56	(1.88–3.48)	< 0.001	1.85	(1.25–2.73)	0.002
SBP, 10 mmHg	0.96	(0.90–1.02)	0.22	1.03	(0.98–1.07)	0.27	1.07	(1.02–1.13)	0.01
Glucose, 10 mg/dl	0.99	(0.96–1.01)	0.36	0.98	(0.97–1.00)	0.09	1.00	(0.98–1.02)	0.87
LDL, 10 mg/dl	1.01	(0.96–1.06)	0.78	0.97	(0.94–1.00)	0.08	1.01	(0.97–1.06)	0.66

P-values from multiple logistic regression models using generalized linear mixed models to account for the center effect (using a random intercept model).

Abbreviations; same as in Table 1.

**Table 3:** Factors associated with taking antiplatelet regimens.

	CM vs AM			AC vs AM			AC vs CM		
	Crude OR (95% CI)		P	Crude OR (95% CI)		P	Crude OR (95% CI)		P
Age, 10 years	1.25	(1.09–1.44)	0.001	1.24	(1.13–1.36)	< 0.001	0.97	(0.86–1.09)	0.59
Male	0.83	(0.62–1.13)	0.24	1.11	(0.90–1.37)	0.31	1.46	(1.12–1.89)	0.005
Onset to arrival									
Within 24 h	0.83	(0.60–1.13)	0.23	0.74	(0.60–0.92)	0.01	0.85	(0.65–1.10)	0.21
Initial NIHSS	0.96	(0.93–0.99)	0.005	0.99	(0.97–1.01)	0.28	1.03	(1.00–1.06)	0.07
Pre-mRS > 1	0.73	(0.48–1.11)	0.14	1.05	(0.78–1.40)	0.75	1.06	(0.75–1.51)	0.73
Mechanisms									
TIA	0.87	(0.52–1.45)	0.60	0.75	(0.54–1.04)	0.09	0.78	(0.48–1.26)	0.31
LAA	1.17	(0.771.76)	0.46	1.91	(1.45–2.53)	< 0.001	1.46	(1.02–2.10)	0.04
SVO	1.82	(1.17–2.82)	0.01	1.23	(0.90–1.67)	0.19	0.57	(0.39–0.84)	0.005
OE/UD (ref)	Ref			Ref			Ref		
History of TIA	1.70	(0.78–3.72)	0.18	1.59	(0.91–2.78)	0.11	1.41	(0.78–2.55)	0.26
History of stroke	1.33	(0.97–1.84)	0.08	1.40	(1.10–1.77)	0.01	0.87	(0.66–1.13)	0.29
History of PAD	0.70	(0.133.82)	0.68	1.29	(0.41–4.01	0.66	1.93	(0.35–10.65)	0.45
History of CAD	1.05	(0.66–1.67)	0.84	1.78	(1.30–2.43)	0.0004	1.90	(1.32–2.74)	0.001
HTN	1.24	(0.84–1.83)	0.28	2.00	(1.54–2.60)	< 0.001	0.99	(0.68–1.42)	0.94
DM	1.02	(0.76–1.39)	0.88	1.41	(1.14–1.75)	0.001	0.98	(0.76–1.26)	0.86
Dyslipidemia	1.70	(1.23–2.34)	0.001	1.28	(1.02–1.60)	0.03	0.79	(0.61–1.04)	0.09
Smoking, current	0.94	(0.67–1.32)	0.73	0.91	(0.72–1.15)	0.44	1.21	(0.90–1.62)	0.20
Prior statin	1.07	(0.76–1.52)	0.68	1.10	(0.87–1.38)	0.43	1.14	(0.86–1.51)	0.38
Prior antihypertensive	1.56	(1.10–2.21)	0.01	2.32	(1.83–2.95)	< 0.001	1.18	(0.85–1.63)	0.33
Prior anti-diabetes	1.16	(0.85–1.58)	0.36	1.45	(1.16–1.81)	0.001	0.91	(0.70–1.18)	0.48
RAD	0.77	(0.55–1.07)	0.11	1.69	(1.36–2.10)	< 0.001	2.23	(1.69–2.95)	< 0.001
Thrombolysis	1.01	(0.57–1.80)	0.97	1.11	(0.74–1.67)	0.61	1.02	(0.62–1.67)	0.94
Antidiabetes Tx	1.02	(0.73–1.42)	0.90	1.53	(1.21–1.92)	0.0003	0.99	(0.75–1.29)	0.91
Antihypertensive Tx	1.27	(0.94–1.72)	0.12	1.35	(1.10–1.66)	0.005	0.93	(0.72–1.21)	0.59
Statin treatment	1.05	(0.72–1.54)	0.78	2.82	(2.15–3.71)	< 0.001	1.88	(1.31–2.69)	0.001
SBP, 10 mmHg	0.97	(0.91–1.03)	0.28	1.02	(0.98–1.06)	0.36	1.06	(1.01–1.11)	0.03
Glucose, 10 mg/dl	0.99	(0.97–1.01)	0.36	1.00	(0.98–1.01)	0.80	1.00	(0.98–1.02)	0.96
LDL, 10 mg/dl	1.01	(0.97–1.05)	0.67	0.99	(0.96–1.02)	0.34	1.00	(0.96–1.04)	0.93

P-values from univariate logistic regression models using generalized linear mixed models to account for the center effect (using a random intercept model).

Abbreviations; same as in Table 1.

### Risk scores of recurrent stroke and antiplatelet regimens

**Figure 1:**
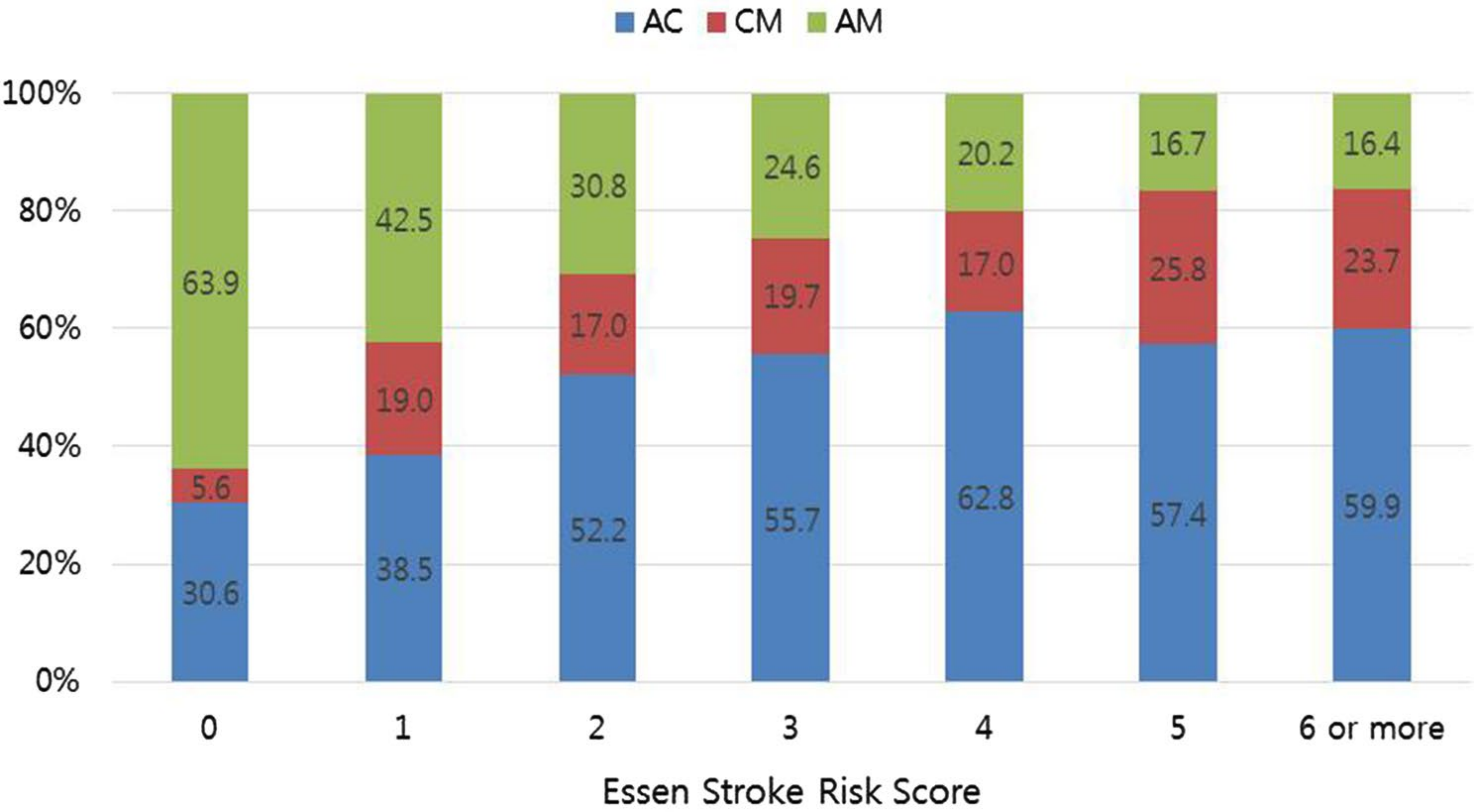
Essen Stroke Risk Scores and antiplatelet strategies.

The likelihood of AC use increased as the ESRS increased (P for trend < 0.001). In contrast, the likelihood of AM use decreased as the ESRS increased (P for trend < 0.001) (Fig. 1). Every 1-point increase in the ESRS was independently associated with a 33% increased likelihood of AC use compared with AM use (adjusted OR 1.33 [1.23–1.45]). When the ESRS was dichotomized, individuals with a higher ESRS (≥ 1 vs 0, ≥ 2 vs 0–1, ≥ 3 vs 0–2) were more likely to have 2- to threefold greater odds of receiving AC compared with receiving AM. Although CM was less frequently used than AM, a 1-point increase in the ESRS was independently associated with a 24% greater odds of receiving CM rather than AM (adjusted OR 1.24 [1.11–1.39], P = 0.0002). However, the relative proportion of AC use versus CM use did not change significantly according to the ESRS (Table 4).

**Table 4:** Essen Stroke Risk Scores and antiplatelet strategies.

	CM (vs AM)			AC (vs AM)			AC (vs CM)	
	Adjusted OR (95% CI)		P	Adjusted OR (95% CI)		P	Adjusted OR (95% CI)	P
ESRS dichotomized								
1 or more vs 0	4.73	(0.99–22.52)	0.05	3.71	(1.70–8.09)	0.001	1.15 (0.20–6.80)	0.88
2 or more vs 0–1	1.47	(0.89–2.42)	0.13	2.64	(1.87–3.74)	< .0001	1.51 (0.89–2.57)	0.12
3 or more vs 0–2	1.64	(1.17–2.31)	0.004	2.01	(1.60–2.53)	< .0001	0.95 (0.70–1.29)	0.74
ESRS, ordinal 1-point increase	1.24	(1.11–1.39)	0.0002	1.33	(1.23–1.45)	< .0001	0.98 (0.88–1.08)	0.65

P-value from multiple logistic regression models using generalized linear mixed models to account for the center effect (using a random intercept model).

Adjusted variables: male, time to admission, initial NIHSS, prestroke disability, TOAST, dyslipidemia, prior statin, and RAD.

Subgroup analyses showed that patients with a higher ESRS were more likely to receive AC rather than AM. However, there was no significant association of ESRS with AC use versus CM use (Table 5).

**Table 5:** Subgroup analysis of the association between Essen Stroke Risk Scores and antiplatelet strategies.

	CM vs AM			AC vs AM			AC vs CM		
	Adjusted OR (95% CI)		P	Adjusted OR (95% CI)		P	Adjusted OR (95% CI)		P
Initial NIHSS									
NIHSS < 4									
ESRS, 1 point increase	1.19	(0.99–1.42)	0.07	1.20	(1.05–1.36)	0.01	0.91	(0.78–1.06)	0.23
ESRS ≥ 1 (vs 0)	NE			6.05	(0.59–62.18)	0.13	NE		
ESRS ≥ 2 (vs 0–1)	1.80	(0.55–5.95)	0.33	2.09	(1.06–4.14)	0.03	0.98	(0.28–3.44)	0.97
NIHSS ≥ 4									
ESRS, 1 point increase	1.25	(1.09–1.44)	0.002	1.42	(1.28–1.58)	< 0.001	1.02	(0.90–1.16)	0.76
ESRS ≥ 1 (vs 0)	NE			3.36	(1.47–7.67)	0.004	NE		
ESRS ≥ 2 (vs 0–1)	1.33	(0.77–2.29)	0.31	2.77	(1.86–4.12)	< 0.001	1.65	(0.92–2.98)	0.09
Onset to admission									
≤ 24 h									
ESRS, 1 point increase	1.16	(0.95–1.41)	0.14	1.34	(1.16–1.54)	< 0.001	1.03	(0.87–1.21)	0.74
ESRS ≥ 1 (vs 0)	NE			8.99	(1.76–46.00)	0.01	NE		
ESRS ≥ 2 (vs 0–1)	1.60	(0.67–3.81)	0.29	3.99	(2.18–7.32)	< 0.001	1.93	(0.72–5.18)	0.19
> 24 h									
ESRS, 1 point increase	1.26	(1.10–1.44)	0.001	1.32	(1.19–1.45)	< 0.001	0.95	(0.85–1.07)	0.44
ESRS ≥ 1 (vs 0)	NE			2.55	(1.05–6.22)	0.04	NE		
ESRS ≥ 2 (vs 0–1)	1.31	(0.73–2.37)	0.37	2.08	(1.38–3.16)	0.001	1.39	(0.75–2.58)	0.30
Relevant arterial diseases									
RAD ( +)									
ESRS, 1 point increase	1.19	(0.98–1.44)	0.08	1.18	(1.03–1.35)	0.01	0.87	(0.74–1.04)	0.12
ESRS ≥ 1 (vs. 0)	0.67	(0.05–8.45)	0.76	7.91	(0.73–85.58)	0.09	3.95	(0.10–152.0)	0.46
ESRS ≥ 2 (vs. 0–1)	1.48	(0.43–5.10)	0.53	2.41	(1.21–4.82)	0.01	1.11	(0.31–3.95)	0.87
No RAD									
ESRS, 1 point increase	1.25	(1.09–1.43)	0.002	1.40	(1.27–1.55)	< 0.001	1.03	(0.92–1.17)	0.59
ESRS ≥ 1 (vs 0)	8.70	(1.05–71.80)	0.04	3.22	(1.42–7.32)	0.005	0.76	(0.08–7.49)	0.81
ESRS ≥ 2 (vs 0–1)	1.38	(0.81–2.36)	0.24	2.62	(1.77–3.89)	< 0.001	1.63	(0.91–2.91)	0.10
TOAST classifications									
LAA									
ESRS, 1 point increase	1.21	(1.01–1.45)	0.04	1.20	(1.05–1.37)	0.01	0.86	(0.73–1.00)	0.05
ESRS ≥ 1 (vs 0)	NE			3.27	(0.44–24.41)	0.25	NE		
ESRS ≥ 2 (vs 0–1)	1.21	(0.45–3.22)	0.71	2.84	(1.54–5.23)	0.001	1.07	(0.41–2.82)	0.89
SVO									
ESRS, 1 point increase	1.23	(0.97–1.55)	0.08	1.50	(1.24–1.81)	< 0.001	1.06	(0.88–1.28)	0.51
ESRS ≥ 1 (vs 0)	NE			2.17	(0.33–14.20)	0.42	NE		
ESRS ≥ 2 (vs 0–1)	0.98	(0.40–2.40)	0.96	2.43	(1.15–5.13)	0.02	2.24	(0.88–5.71)	0.09
OE/UD									
ESRS, 1 point increase	1.23	(0.97–1.56)	0.09	1.35	(1.15–1.58)	0.0003	1.07	(0.85–1.35)	0.54
ESRS ≥ 1 (vs 0)	NE			20.24	(2.31–177.2)	0.01	NE		
ESRS ≥ 2 (vs 0–1)	1.48	(0.49–4.43)	0.48	2.18	(1.06–4.49)	0.03	1.66	(0.48–5.74)	0.42

NE; non-estimable, other abbreviations; same as in Table 1.

Adjusted variables: male, time to admission, initial NIHSS, prestroke disability, TOAST, dyslipidemia, prior statin, and RAD.

P-values from multiple logistic regression models using generalized linear mixed models to account for the center effect (using a random intercept model).

### Outcomes

The mean follow-up duration was 364 days. The primary outcome event, a composite of stroke, MI, and all-cause mortality, occurred in 164 patients, and its one-year cumulative rate was 9.6%. Based on a crude analysis, the one-year event rates of the primary composite outcome were numerically higher in the AM group than the CM and the AC group (12.1% vs 8.7% vs 8.8%, respectively)(P = 0.11) (Table 6). When stratified into 3 ESRS categories, the 1-year event rates of the primary composite outcome were significantly higher in the AM group than the CM and the AC group among patients with ESRS 4 or more (22.8% vs 6.6% vs 11.5%, respectively) (P < 0.001), whereas among patients with ESRS 0–1 or 2–3, the primary composite outcomes were not significantly different among the 3 groups (Table 6). The Cox proportional hazard regression analysis revealed that compared with AM, AC was independently associated with the reduction of the composite of stroke, MI, and all-cause mortality (adjusted HR 0.66, [0.46–0.95], P = 0.02) (Table 7). In addition, associations of antiplatelet regimen with the primary outcome event were significantly modified by the ESRS categories (Table 7). Among patients with ESRS 4 or more, AC and CM significantly reduced the risk of primary outcome events compared with AM, whereas among patients with ESRS 0–1 or 2–3, no significant difference was observed among 3 treatment regimens.

**Table 6:** One-year event rates according to antiplatelet regimen.

	AM group	CM group	AC group	P*
All patients, N	593	456	1299	
Primary outcome, n (%)	48 (12.1)	29 (8.7)	87 (8.8)	0.11
Stroke, n (%)	17 (4.5)	17 (5.0)	47 (4.9)	0.92
ESRS categories				
ESRS 0–1	97	35	78	
Primary outcome, n (%)	3 (6.5)	1 (4.8)	4 (6.0)	0.96
Stroke, n (%)	3 (6.5)	0	4 (6.0)	0.52
ESRS 2–3	317	217	634	
Primary outcome, n (%)	14 (6.7)	17 (11.6)	33 (6.7)	0.16
Stroke, n (%)	4 (1.9)	9 (5.9)	18 (3.8)	0.13
ESRS 4 or more	179	204	587	
Primary outcome, n (%)	31 (22.8)	11 (6.6)	50 (11.5)	< 0.001
Stroke, n (%)	10 (8.0)	8 (4.7)	25 (5.8)	0.53

*Calculated by the log-rank test.

**Table 7:** HR (95% CI) for a primary composite outcome in all patients and subgroups according to the ESRS categories: results of the Cox proportional hazard regression analysis.

	Crude HR (95% CI)	P	P_int_	Adjusted HR (95% CI)	P	P_int_
All patients						
AM group	Ref			Ref		
CM group	0.70 (0.44–1.11)	0.13		0.70 (0.43–1.11)	0.13	
AC group	0.70 (0.49–0.99)	0.04		0.66 (0.46–0.95)	0.02	
ESRS categories			0.007			0.006
ESRS 0–1						
AM (ref)	Ref			Ref		
CM	0.81 (0.08–7.79)	0.86		0.68 (0.07–6.55)	0.74	
AC	1.10 (0.25–4.90)	0.90		0.90 (0.20–4.04)	0.89	
ESRS 2–3						
AM (ref)	Ref			Ref		
CM	1.69 (0.84–3.44)	0.14		1.98 (0.97–4.05)	0.06	
AC	1.00 (0.53–1.86)	0.99		1.04 (0.56–1.96)	0.90	
ESRS 4 or more						
AM (ref)	Ref			Ref		
CM	0.27 (0.13–0.53)	0.0002		0.30 (0.15–0.60)	0.001	
AC	0.44 (0.28–0.69)	0.0004		0.47 (0.30–0.74)	0.001	

Adjusted variables: age, sex, NIHSS, ischemic events subtype (TOAST including TIA), RAD, prior statin use, and ESRS.

P-values from the shared frailty model to account for the center effect.

P_int_: P-value for the interaction.

## Discussion

In this study, over 2,300 patients who experienced acute cerebral ischemia while taking aspirin were enrolled in a prospective, multicenter stroke registry in South Korea, and approximately half of patients were treated with a combination of AC. In contrast, less than 1 of 5 patients was treated with CM, and this frequency was even lower than that for AM use.

We found that the use of a more potent antiplatelet regimen (i.e., AC combination) increased as the ESRS increased. The tendency to prefer AC in high-risk patients was observed for other factors, such as initial NIHSS score (≤ 4 vs > 4), onset to admission (≤ 24 h vs > 24 h), RAD, and TOAST classifications. Accordingly, stroke physicians appear to intuitively select a potent antiplatelet regimen in patients who experience breakthrough acute cerebral ischemia while on aspirin and have a high risk of future vascular events. However, our study did not imply that the choice of AC following aspirin failure would be correct.

Our study also shows that factors associated with vascular status (indicated by prior non-cerebral vascular diseases and cerebral arterial steno-occlusion) as well as traditional risk factors might affect the selection of antiplatelet regimen in these patients. The combination of AC was more likely selected than AM in patients with high atherosclerotic burdens, such as older age, history of vascular diseases, or large artery disease. The preference of dual antiplatelet therapy over monotherapy is presumably extrapolated from the results of coronary clinical trials^14,15^ and indirect evidence from trials on stroke populations with severe atherosclerosis^2,16^. In contrast, for patients with SVO, the AC regimen is less frequently used, which is presumably influenced by the Secondary Prevention of Small Subcortical Strokes (SPS-3) trial results, which showed that AC did not reduce recurrent stroke compared with AM but did significantly increase the risk of major bleeding and death^17^. In patients with breakthrough stroke while on aspirin, CM might be a more reasonable alternative to aspirin, which needs to be confirmed by randomized trials.

The observation that AC use was substantially more common (approximately 3 times more frequent) than CM use in our study was contrary to expectations because the MATCH trial and a prior meta-analysis indicated that CM was comparable to AC for preventing recurrent vascular events and safer in terms of risk of major or intracranial bleeding^18,19^. In the Clopidogrel vs. Aspirin in Patients at Risk of Ischemic Events (CAPRIE) trial, patients with a history of prior vascular disease had a high rate of subsequent ischemic events and the absolute benefit of clopidogrel over aspirin seemed to be amplified in such high-risk patients^20^. In addition, as the antiplatelet effect of aspirin usually lasts several days, clopidogrel administration immediately after breakthrough cerebral ischemia while on aspirin is expected to have the effect of short-term dual antiplatelet therapy^21^.

However, CM might be less frequently used because evidence for the use of clopidogrel is limited in the context of acute ischemic stroke^22^, and a recent study suggested that the antiplatelet regimen in the first few days after ischemic stroke should include aspirin^23^. Other antiplatelet drugs did not reduce the risk or severity of early recurrent stroke^23^. However, as the combination therapy was only supported in patients with symptomatic high-grade intracranial stenosis or acute minor stroke or TIA over 3 months in randomized trials^3,24^, our results regarding physicians’ preference for AC after aspirin failure were somewhat unexpected. Therefore, these findings may address the need for a randomized clinical trial to explore the optimal antiplatelet strategy in aspirin failure.

Our results indicate that physicians seem to select an antiplatelet regimen based on an intuitive estimate of future vascular event risk. Patients with ESRSs between 0 and 1, which is equivalent to a presumed low risk of recurrent stroke, were most frequently treated with AM. However, physicians advocated AC in more than half of patients with an ESRS of 2 or more points. Patients with 1 or more point had a 3.7-fold higher likelihood of being treated with AC than AM compared those with 0 points. Also, for every 1-point increase, AC (OR 1.33 [1.23–1.45]) and CM (OR 1.24 [1.11–1.39] were more likely to be used compared with AM.

We found that patients with a higher ESRS score had a greater risk of recurrent stroke or vascular events^8^. The combination of ER-DP and aspirin or clopidogrel might be particularly beneficial in preventing recurrent stroke in moderate- to high-risk patients compared with aspirin alone as assessed by the stroke risk models^25,26^. In a post hoc analysis of the European Stroke Prevention Study 2, aspirin plus ER-DP reduced the risk of annual stroke by 30% in the high-risk group compared with aspirin alone as assessed by the Framingham score, but this result was not observed in the low-risk group^25,26^. These preferential benefits of the combination therapies in high-risk population were reproduced in our study. While AC did not reduce the risk of the composite of stroke, MI, and all-cause mortality at 1 year among patients with ESRS of 0–1, it did reduce the risk by 56% among patients with ESRS 4 or more compared with AM. These results suggest that a risk prediction scale, such as the ESRS, might help clinicians stratify individual patients according to the risk of subsequent stroke, identify patients who will benefit more from aggressive medications, and tailor treatments, especially in cases lacking clear evidence. However, as our study was not designed to evaluate the efficacy of antiplatelet regimen, the results should be interpreted with caution. Further studies are warranted.

Interestingly, our study found that although approximately 80% of the study population presented with hypertension, only approximately 50% of the patients were receiving antihypertensive therapy at admission. In contrast, the proportion receiving statin during hospitalization was much higher than that being diagnosed with dyslipidemia at discharge. These results seemed to reflect recent trends for in-hospital treatment of acute ischemic stroke. Statin therapy has been widely applied to patients with acute ischemic stroke for secondary prevention of stroke^27,28^, while there is no evidence that antihypertensive therapy during acute periods of ischemic stroke could improve clinical outcomes^29–31^..

‘Aspirin failure’ is defined as breakthrough stroke or TIA in patients taking aspirin. The reasons for aspirin failure are unclear, although insufficient platelet inhibition by aspirin is considered an important reason. Several factors are reported to be associated with insufficient platelet inhibition by aspirin, including chronic kidney disease^32^, body weight^33^, and drug-drug interactions^34^. However, our study was limited because we did not investigate the mechanisms of aspirin failure, such as aspirin resistance, on the platelet function test. Further studies should be performed to investigate the optimal antiplatelet regimen based on the mechanism of aspirin failure.

Our study has several limitations. First, it was presented the weak points that are inherent to retrospective, single-nation studies. Therefore, the study results should be generalized with caution. Also, information on the patients’ characteristics, risk factors, medication, and further laboratory findings was limited. Moreover, we did not assess platelet function for this study and did not identify patients with insufficient platelet inhibition. However, the data were obtained from 14 stroke centers located nationwide and 74 neurologists independently determined the antiplatelet regimen; thus, the data had certain strengths. Because of lack of commercial availability of ER-DP in clinical practice, we were not able to analyze data on ER-DP, which is widely used in other countries. In addition, we could not confirm whether the treatment regimen at discharge continued without change until the end of follow-up. Third, we did not capture any bleeding events related to antithrombotic therapy. Although more general and important safety outcomes of all-cause mortality were analyzed, the results should be interpreted with caution. Finally, the inability to exclude reverse causation and residual confounders related to imbalances in baseline characteristics do not allow us to accept the study results as conclusive. The prescription of antithrombotic drugs may have been affected many unmeasured factors other than those considered in our study, including physician personal experience, medication availability, possibly previous patient experience with the drugs, compliance with multiple medications, patient preference, and concerns about risk factors. Therefore, the results of our study should be interpreted with caution.

In conclusion, a combination of AC was most preferentially selected for over half of the patients with breakthrough stroke while on aspirin in Korea. A higher ESRS indicated higher risk of future vascular events and was associated with an increased use of the combination AC therapy. These results suggest that Korean physicians prefer a more potent antiplatelet strategy in patients with aspirin failure and higher risk of future vascular events. In addition, a combination of aspirin and clopidogrel might lead to more substantial reductions in the 1-year vascular events among patients with higher ESRSs. Future trials are warranted to identify the optimal antiplatelet regimen according to the risk of subsequent vascular events in patients who have strokes while on aspirin.

## Supplementary information

Supplementary file1

## Data Availability

The datasets generated and/or analyzed during the current study are available upon reasonable request from the corresponding author by email: braindoc@snu.ac.kr.
