# Supplementary material for: Risk of recurrent stroke and antiplatelet choice in breakthrough stroke while on aspirin
Source: Sci Rep. 2020 Oct 7;10:16723. doi: 10.1038/s41598-020-73836-0 (PMC7541489; doi:10.1038/s41598-020-73836-0)
Supplement: Supplementary file 1 — Supplementary file1 [file 41598_2020_73836_MOESM1_ESM.docx]

**Supplemental Materials**

**Title:** Risk of Recurrent Stroke and Antiplatelet Choice in Breakthrough Stroke While on Aspirin

Joon-Tae Kim, MD, PhD,^a^ Beom Joon Kim, MD, PhD,^b^ Jong-Moo Park, MD, PhD,^c^ Soo Joo Lee, MD, PhD,^d^ Jae-Kwan Cha, MD, PhD,^e^ Tai Hwan Park, MD, PhD,^f^ Kyung Bok Lee, MD, PhD,^g^ Jun Lee, MD, PhD,^h^ Keun-Sik Hong, MD, PhD,^i^ Byung-Chul Lee, MD, PhD,^j^ Dong-Eog Kim, MD, PhD,^k^ Jay Chol Choi, MD, PhD,^l^ Jee-Hyun Kwon, MD, PhD,^m^ Dong-Ick Shin, MD, PhD,^n^ Sung Il Sohn, MD, PhD,^o^ Ji Sung Lee, PhD,^p^ Juneyoung Lee, PhD,^q^ Hee-Joon Bae, MD, PhD.^b^

Supplemental Methods

Supplemental Table: I-III

Supplemental Figure: I

Supplemental Figure I. Selection of the study population

30,135 patients with acute ischemic stroke or TIA in CRCS-5 registry (Apr 2008-Mar 2014)

3,140 non-cardioembolic stroke with prior aspirin use

2,348 Patients included in the primary analysis

792 excluded

Discharge anticoagulation (n=446)

Antiplatelets other than aspirin/clopidogrel (n=346)

26,995 excluded

Not admitted within 7 d of stroke onset (n=1,134)

Cardioembolic stroke (n=6,456)

Prior non-antiplatelet users (n=16,880)

Prior non-aspirin users (n=2,525)

Supplemental Table I. Essen Stroke Risk scores

|  | Score |
| --- | --- |
| Age >75yr | 2 |
| Age 65-75yr | 1 |
| HTN | 1 |
| DM | 1 |
| Previous MI | 1 |
| Other CV disease (except MI and AF) | 1 |
| PAD | 1 |
| Current or recent smoking | 1 |
| Previous TIA or IS in addition to qualifying event | 1 |

Supplemental Table II. General subject characteristics, including other monotherapy

|  | Aspirin mono | Other mono | Clopidogrel mono | Aspirin + Clopidogrel | P_1_ | P_2_ |
| --- | --- | --- | --- | --- | --- | --- |
| N | 593 | 346 | 456 | 1299 |  |  |
| Age | 66±12 | 69±11 | 70±11 | 69±11 | <0.001 | 0.003 |
| Male | 334 (56.3) | 175 (50.6) | 252 (55.3) | 769 (59.2) | 0.03 | 0.09 |
| Time to admission  within 24 h | 403 (68.0) | 126 (36.4) | 285 (62.5) | 783 (60.3) | 0.02 | 0.18 |
| Prestroke mRS >1 | 92 (15.5) | 71 (20.5) | 74 (16.2) | 198 (15.2) | 0.12 | 0.06 |
| Baseline NIHSS (med, IQR) | 2 (0, 5) | 3 (1, 5) | 2 (1, 4) | 3 (1, 5) | 0.26 | 0.15 |
| Presenting event |  |  |  |  | 0.001 | 0.02 |
| TIA | 116 (19.6) | 46 (13.3) | 58 (12.7) | 171 (13.2) |  |  |
| Ischemic stroke | 477 (80.4) | 300 (86.7) | 398 (87.3) | 1128 (86.8) |  |  |
| TOAST (except TIA) |  |  |  |  | <0.001 | <0.001 |
| LAA | 196 (41.1) | 155 (44.8) | 159 (39.9) | 608 (53.9) |  |  |
| SVO | 132 (27.7) | 87 (25.1) | 161 (40.5) | 273 (24.2) |  |  |
| OE | 15 (3.1) | 6 (1.7) | 6 (1.5) | 20 (1.8) |  |  |
| UD | 134 (28.1) | 52 (15.0) | 72 (18.1) | 227 (20.1) |  |  |
| History of TIA | 18 (3.0) | 15 (4.3) | 21 (4.6) | 64 (4.9) | 0.32 | 0.36 |
| History of stroke | 147 (24.8) | 148 (42.8) | 165 (36.2) | 391 (30.1) | <0.001 | <0.001 |
| History of PAD | 5 (0.8) | 5 (1.4) | 2 (0.4) | 13 (1.0) | 0.51 | 0.51 |
| History of CAD | 63 (10.6) | 48 (13.9) | 55 (12.1) | 240 (18.5) | <0.001 | 0.14 |
| CAD except MI | 36 (6.1) | 32 (9.2) | 38 (8.3) | 153 (11.8) | 0.001 | 0.09 |
| MI | 27 (4.6) | 16 (4.6) | 17 (3.8) | 87 (6.7) | 0.05 | >0.99 |
| HTN | 450 (75.9) | 297 (85.8) | 393 (86.2) | 1126 (86.7) | <0.001 | <0.001 |
| DM | 212 (35.8) | 172 (49.7) | 205 (45.0) | 583 (44.9) | <0.001 | <0.001 |
| Dyslipidemia | 186 (31.4) | 131 (37.9) | 224 (49.1) | 504 (38.8) | <0.001 | 0.05 |
| Smoking | 160 (27.0) | 70 (20.2) | 111 (24.3) | 328 (25.3) | 0.13 | 0.02 |
| Prior statin | 159 (26.8) | 104 (30.1) | 138 (30.3) | 408 (31.4) | 0.25 | 0.29 |
| Prior antihypertensive | 396 (66.8) | 274 (79.2) | 366 (80.3) | 1082 (83.3) | <0.001 | <0.001 |
| Prior antidiabetics | 173 (29.2) | 149 (43.1) | 179 (39.3) | 491 (37.8) | <0.001 | <0.001 |
| RAD (>50%) | 186 (31.4) | 131 (37.9) | 125 (27.4) | 542 (41.7) | <0.001 | 0.05 |
| Laboratory findings |  |  |  |  |  |  |
| Glucose (mg//dl) | 141±70 | 144±65 | 145±72 | 142±64 | 0.77 | 0.50 |
| LDL (mg/dl) | 104±36 | 104±37 | 109±36 | 103±33 | 0.02 | 0.92 |
| SBP (mmHg) | 148±27 | 146±25 | 145±25 | 149±25 | 0.01 | 0.28 |
| Thrombolysis | 41 (6.9) | 13 (3.8) | 33 (7.2) | 94 (7.2) | 0.13 | 0.06 |
| Hospital treatment |  |  |  |  |  |  |
| Antihypertensive | 279 (47.0) | 203 (58.7) | 265 (58.1) | 728 (56.0) | <0.001 | 0.001 |
| Antidiabetics | 157 (36.5) | 141 (40.8) | 167 (36.6) | 453 (34.9) | <0.001 | <0.001 |
| Statin | 451 (76.1) | 283 (81.8) | 368 (80.7) | 1156 (89.0) | <0.001 | 0.04 |
| ESRS (med, IQR) | 3 (2, 4) | 4 (3, 5) | 3 (2, 5) | 3 (2, 4) | <0.001 | <0.001 |
| 1 or more | 570 (96.1) | 340 (98.3) | 454 (99.6) | 1288 (99.2) | <0.001 | 0.08 |
| 2 or more | 496 (83.6) | 319 (92.2) | 421 (92.3) | 1221 (94.0) | <0.001 | <0.001 |

P_1_: comparisons among groups; and P_2_: comparison between aspirin monotherapy and other monotherapy

|  |
| --- |

Supplemental Table III. Detailed antiplatelet strategies after new ischemic stroke

|  | N | % |
| --- | --- | --- |
| Aspirin monotherapy | 593 | 22.0 |
| Clopidogrel monotherapy | 456 | 16.9 |
| Aspirin + clopidogrel combination | 1299 | 48.2 |
| Others | 346 | 12.8 |
| Other monotherapy (neither aspirin nor clopidogrel) | 61 | 2.3 |
| Aspirin + non-clopidogrel combination | 222 | 8.0 |
| Other combination (neither aspirin nor clopidogrel) | 31 | 1.2 |
| Triple or more combination | 32 | 1.2 |
| Total | 2694 | 100 |
